# Consideration of Anesthesia Techniques for Deep Brain Stimulation Implantation in the Treatment of Drug-Resistant Epilepsy: A Narrative Review

**DOI:** 10.3390/biom15060784

**Published:** 2025-05-28

**Authors:** Alan D. Kaye, Benjamin Esneault, Shreya Deshpande, Joseph Wentling, Shahab Ahmadzadeh, Pooja Potharaju, Sahar Shekoohi

**Affiliations:** 1 Departments of Anesthesiology and Pharmacology, Toxicology and Neurosciences, Louisiana State University Health Sciences Center at Shreveport, Shreveport, LA 71103, USA; 2 School of Medicine, Louisiana State University Health Sciences Center at Shreveport, Shreveport, LA 71103, USA; 3 SS Institute of Medical Sciences and Research Centre, Davangere 577003, India; shreya.desh23@gmail.com; 4 School of Medicine, Louisiana State University Health Sciences Center at New Orleans, New Orleans, LA 70112, USA; jwentl@lsuhsc.edu; 5 Department of Anesthesiology, Louisiana State University Health Sciences Center at Shreveport, Shreveport, LA 71103, USA

**Keywords:** DBS, epilepsy, anesthesia, MER, ANT, CMT

## Abstract

Epilepsy is a neurological disorder characterized by recurrent, unprovoked seizures, affecting millions worldwide. While anti-seizure medications serve as first-line treatment, approximately one-third of patients develop drug-resistant epilepsy (DRE), necessitating alternative interventions. Deep brain stimulation (DBS) has emerged as a promising therapy for DRE, particularly for patients who are ineligible for resective surgery. DBS involves stereotactic implantation of electrodes into target brain regions, such as the anterior nucleus of the thalamus (ANT), centromedian nucleus (CMT), and hippocampus (HC), to modulate aberrant neural activity and to reduce seizure frequency. Anesthesia plays a critical role in DBS implantation, influencing both patient safety and procedural success. The choice of anesthetic technique must balance patient comfort with the preservation of neurophysiological signals used for intraoperative electrode localization. A well-chosen anesthetic strategy can enhance the efficacy of electrode placement by minimizing patient movement and preserving critical neurophysiological signals for real-time monitoring. This precise targeting enhances safety via a reduction in perioperative risks and an improvement in long-term seizure control. Anesthetic considerations in epilepsy patients differ from those in movement disorders due to variations in their nuclei targets during DBS. Despite the increasing use of DBS for epilepsy following its FDA approval in 2018, research on anesthetic effects specific to this population remains limited. This narrative review, therefore, examines anesthetic approaches, pharmacological implications, potential complications, and evolving methods for DBS implantation in epilepsy patients, highlighting new insights and unique considerations in this population. Understanding these factors is essential for optimizing surgical outcomes and improving the safety and efficacy of DBS in epilepsy treatment.

## 1. Introduction

Epilepsy is defined as the recurrence of multiple, unprovoked seizures. Seizures are brief, excessive discharges in the brain that occur from abnormal neuron propagation. This abnormal communication between neurons can be caused by diminished neuron inhibition, excessive excitation, or a combination of the two [1]. According to a Swedish longitudinal cohort study, people with epilepsy have a 14-fold increase in all-cause mortality. This increase can be attributed both directly to epilepsy, in cases of status epilepticus for instance, and indirectly to causes such as accidents, drowning, and suicide [2].

Treatment has been shown to be effective, with approximately 60–70% of patients able to achieve seizure freedom with proper diagnosis and treatment. First-line treatment for most epilepsy patients is a regimen of anti-seizure medications, or AEDs [1]. However, almost one-third of patients continue to have seizures despite optimized medical treatment and are consequently diagnosed with drug-resistant epilepsy (DRE) [3]. Resective surgery is considered to be a safe and effective option for many patients with DRE; however, approximately half of DRE patients are not deemed suitable candidates for the procedure [3]. As a result, alternative treatments have arisen to provide epilepsy patients with relief, including deep brain stimulation (DBS), vagus nerve stimulation (VNS), and responsive neurostimulation (RNS) [3].

DBS is a neuromodulatory treatment most associated with the treatment of movement disorders, such as Parkinson’s disease (PD). The mechanism of DBS varies depending on the electrical parameters indicated and the targeted brain area. In epilepsy patients, DBS is believed to result in the disruption of seizure propagation or the alteration of seizure threshold [3]. The DBS procedure involves the implantation of electrodes during stereotaxic surgery. During the procedure, electrophysiological guidance with intraoperative microelectrode recordings (MERs) and macrostimulations are used to localize and confirm the target nuclei [4]. For example, a study investigating ANT stimulation for drug-resistant epilepsy indicated discernable features of the ANT using MERs [5]. These unique features include a mean spiking rate of 7.52 Hz ± 6.9 Hz and maximum local neural activity consisting of elevated spiking and bursting at the ANT–mammillothalamic tract junction, indicating that this junction is the optimal target for seizure reduction. The study also showed that both theta (4–8 Hz) and alpha band power (9–12 Hz) were negatively correlated with distance to the ventral ANT border [5]. DBS is typically performed under local and general anesthesia with propofol, a GABA-A agonist, being the most widely used anesthetic for sedation and general anesthesia [6].

In movement disorders, including Parkinson’s and dystonia, the type and depth of anesthesia have been shown to impact MERs during DBS surgery, which is an important safety and efficacy consideration, as proper nuclei localization is dependent on MERs [6]. However, research concerning these anesthetic effects in epilepsy patients specifically is currently emerging, largely due to the fact that DBS is relatively newly used in epilepsy patients and was not approved by the FDA to treat epilepsy until 2018 [3]. Anesthetic effects can vary depending on the nucleus targeted because spontaneous firing rates and patterns vary between nuclei [4]. In epilepsy patients, DBS surgery primarily targets the anterior thalamic nucleus (ANT). Two other targets include the centromedian nucleus (CMT) and hippocampus (HC) [3]. These nuclei targets are unique to DBS in epilepsy; therefore, anesthetic techniques can vary from those commonly used in other disorders.

The present investigation, therefore, explores anesthetic approaches, pharmacological considerations, complications, and emerging trends of DBS implantation surgery in epilepsy patients. This narrative review is a conceptual overview that aims to clarify fundamental concepts, provide context, and highlight the innovations of a field of medicine that is relevant and evolving. Studies included in this review include clinical trials, meta-analyses, case studies, and systematic reviews, most of which are recent and part of a vast array of emerging research on this topic.

## 2. Anesthetic Approaches

**Figure 1:**
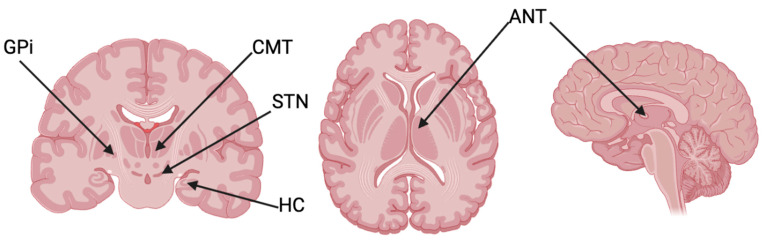
Nuclei targets of DBS for epilepsy (ANT, CMT, HC) shown alongside the DBS targets of PD (STN) and dystonia (GPi).

The selection of anesthetic techniques in DBS implantation is generally based on a number of considerations including patient age, comorbidities, and surgical site [7]. For epilepsy patients, all of the literature regarding the neurostimulation of the ANT indicates that general anesthesia is the method of choice versus local anesthesia or a combination of general and local anesthesia [8,9,10,11]. According to a meta-analysis focusing on the most optimal methods for targeting the ANT, 161 of 162 (99.4%) epilepsy patients were given general anesthesia across seven different studies [12]. This contrasts with anesthetic approaches of DBS in movement disorders, such as Parkinson’s and dystonia, which target the subthalamic nucleus (STN) and globus pallidus internus (GPi), respectively [6]. Local anesthesia is traditionally used in DBS implantation for these disorders because it allows for a more reliable MER to locate the target nucleus [7]. The nuclei targets for epilepsy, PD, and dystonia are shown in Figure 1.

The choice of anesthetic agents and techniques varies based on each individual institution’s practice. Basic physiological monitoring is standard, but in DBS cases anesthesiologists often add depth of anesthesia monitoring (e.g., BIS index) to fine tune the sedation level [13]. Maintaining a BIS in the 40–60 range can ensure that the patient is adequately unconscious yet not so deeply anesthetized that neural signals are obliterated. The literature on anesthetics used in epilepsy cases is limited compared to the more prominent use of DBS in movement disorders. While most of the literature refers solely to the use of “general anesthesia”, there are a few cases that explicitly mention the drugs of choice used in this procedure. A 2018 Dutch study showed that propofol can be safely used for general anesthesia without major influences on MERs during ANT-DBS implantation in epilepsy patients [14]. Retrospective analysis from this study showed that propofol was successfully used in all 23 of the epilepsy patients treated. In the case presented, optimal MERs occurred when propofol was given in a dosage of 8 mg/kg/h. The procedure had no complications, and the patient had a 30% reduction in seizure frequency.

LGS is a treatment-resistant form of childhood-onset epilepsy consisting of multiple seizure types that encompass approximately 1–2% of all patients with epilepsy. In this trial, all 19 patients were given a modified anesthetic regimen combining intravenous remifentanil (0.1–0.3 μg/kg/min) with inhalational isoflurane (0.5–0.7%), which allowed for intraoperative simultaneous EEG recordings from the thalamus and scalp. This anesthetic regimen was chosen because it was determined that it does not suppress epileptic discharges in patients with LGS and produces a scalp EEG similar to what is seen in light natural sleep [15].

## 3. Pharmacological Considerations

Anesthetic agents are crucial components of DBS surgery for a multitude of reasons. Ensuring adequate intraoperative monitoring of MERs dictates the outcomes and success rate of the procedure. The anesthetic used must have minimum impact on the neuronal firing and must not interfere with the MER readings in the lead impaction phase for brain mapping and accurate detection of the epileptiform focus [13]. The anesthetic strategy employed to successfully complete DBS surgery is the “asleep-awake-asleep” technique, which is achieved using Dexmedetomidine. The various strategies used have been studied for the pediatric population and patients with movement disorders, dystonia, and Parkinson’s disease, but there is no clinically relevant data for patients with drug-resistant epilepsy.

Propofol, a direct GABA activator, is a widely used drug for DBS surgery. It has a rapid onset and short duration of action. Its effects on MERs have been extensively studied in patients with dystonia and PD but minimally studied in epileptics. Its effect varies with each brain nucleus targeted. It decreases neuronal firing rates in Globus Pallidus internus (GPi) and has a lesser effect on the STN [13]. The reason for this could be attributed to the presence of more GABA input in the GP and Globus Pallidus externus (GPe) than the STN, which is glutaminergic [13]. Propofol has also been shown to mask High-Frequency Oscillations (HFOs) from epileptiform foci, such as the entorhinal cortex, which hinders their identification intraoperatively [16]. Benzodiazepines are rarely used for DBS surgery as they directly interfere with and abolish MER. Volatile anesthetics such as Sevoflurane, Enflurane, and Desflurane are a class of drugs with an incompletely understood, complex mechanism of action.

There has been restricted use of these drugs in DBS surgery, and the existing literature is mostly limited to use in patients with dystonia and PD. Since there have been very few studies on the use of these drugs with small sample sizes, they should be avoided in favor of better alternatives. Opioids such as fentanyl and remifentanil have not been used singly but have been used in combination with other sedatives or general anesthetics; thus, their effect on MERs is not well documented. The most suitable anesthetic for DBS surgery is said to be Dexmedetomidine [13].

The reason for its ideality is related to the mechanism of action being a selective alpha 2 receptor agonist, thus having no effect on GABA and GABA receptors and causing minimal effect on MER, making it the most appropriate drug for brain mapping [6]. It is known to cause hypotension and tachycardia in PD patients [17], but these complications can easily be managed intraoperatively. High-dose dexmedetomidine can cause the abolition of the MER [18]. Each drug’s effects on MER, benefits, and drawbacks have been compiled into Table 1.

## 4. DBS Complications

DBS is an effective treatment strategy in patients with refractory epilepsy who respond poorly to anti-seizure medications. The long-term safety and efficacy profile is still a subject of ongoing investigation. Compared to the safety profile of DBS performed for PD and movement disorders, there are limited data about DBS for epilepsy. The most common intraoperative complications encountered during DBS surgery are airway difficulty, respiratory depression, bradycardia, and hypoxemia, all of which can be reasonably explained as side effects of anesthetics used for the procedure [13]. In some cases, there may be an unplanned conversion to general anesthesia. A notable side effect of DBS surgery when performed in a semi-sitting position is venous air embolism [13].

The Stimulation of Anterior Nucleus of Thalamus for Treatment of Epilepsy (SANTE) trial was a multicenter, randomized double-blind clinical trial performed to evaluate the efficacy and safety of DBS for refractory epilepsy. Upon the follow up of patients enrolled in this trial at 13 months post-op, most of the adverse effects seen were due to the implantation of electrodes, namely, paresthesia (18.2%), implant site pain (10.9%), and infection (9.1%) [9]. Anesthetic considerations for patients with seizure disorder include minimizing drug–drug interactions to precipitate seizure activity. Other known side effects include electrode migration, lead tract fibrosis, peri-electrode edema, etc., which were less commonly encountered [23]. The specific adverse effects of anesthesia for DBS in this patient population is a point of ongoing study. The SANTE study group conducted follow up at 7 and 10 years post-stimulation to assess the incidence of sudden unexpected death in epilepsy (SUDEP). They found a 75% reduction in median seizure frequency and a statistically significant reduction (71%) in the seizure type, from focal to bilateral tonic–clonic [24]. The SUDEP rate was 2 deaths in 1000 person years [24]. The quality of life in epilepsy measure showed statistical improvement, and 16% of the subjects enrolled were seizure free for 6 months [25]. Psychiatric side effects such as depression, suicide, apathy, and cognitive decline are reported post-DBS. The SANTE trial initially reported higher rates of depression and memory deficits, but the follow-up study of the SANTE participants did not report depression or cognitive decline [23]. Apathy post-DBS with STN stimulation has been reported in patients with epilepsy. The etiology has been hypothesized to be due to the influence of STN-DBS on the limbic system due to stimulus diffusion [23]. Compared to the general population, epileptics are at an increased risk for comorbidities, such as depression and obesity. Thus, careful patient selection for neurostimulatory procedures must be performed to alleviate the risk and improve outcomes [23].

Given the existing literature, the long-term treatment profile and efficacy of DBS for epilepsy indicate favorable outcomes with minimal adverse effects. A double-blind study with 110 patients performed to assess outcomes of a bilateral ANT stimulation as a treatment option for epilepsy reported a 29% decrease in seizures and a low complication rate, indicating a favorable outcome for patients with medically refractory seizures [9]. Robust, large, multicenter clinical trials are the need of the hour to assess the real-world disease and symptom burden and response to DBS. As research on this intriguing topic advances, DBS may become a superlative treatment modality for refractory epilepsy.

## 5. Emerging Trends

Recent years have seen a notable shift in anesthetic practices for DBS surgery, particularly as it is applied to epilepsy. Traditionally, DBS lead implantation was performed with patients awake under local anesthesia to allow intraoperative neurophysiological monitoring [26]. However, improvements in imaging and surgical technology now enable precise electrode placement without awake neurophysiological testing.

Frameless stereotactic systems and intraoperative MRI guidance have increased the use of general anesthesia (GA) for “asleep DBS”, even in cases that previously required awake techniques [4]. This trend is also patient driven, as many individuals (especially those with anxiety or severe symptoms) prefer to avoid awake neurosurgery [26]. As a result, “asleep DBS” is becoming more common for epilepsy, improving patient comfort and expanding access to those who might not tolerate awake procedures [27].

Another emerging trend is the exploration of new anesthetic agents and tailored regimens that minimize interference with electrophysiological recordings. Propofol-based total intravenous anesthesia (TIVA) has gained popularity for DBS in epilepsy due to its short-acting profile and limited impact on neuronal firing patterns. Notably, a 2018 report by Bos et al. demonstrated that propofol can be safely used for ANT-DBS under GA without compromising MER signals [14]. In that case, the initial use of sevoflurane completely suppressed thalamic neuronal activity, but switching to propofol restored the characteristic burst firing in the ANT.

These findings underscore propofol’s role as a GA agent that preserves neural signals, in contrast to volatile anesthetics, like sevoflurane, which can markedly dampen neuronal activity. Consequently, many centers favor TIVA (propofol ± short-acting opioid) for asleep DBS in epilepsy. Meanwhile, alternative sedation techniques are being investigated to balance patient comfort with the need for neurophysiological data. Dexmedetomidine, a sedative that mimics natural sleep, has been used as an adjunct in DBS anesthesia to provide moderate sedation without abolishing MERs; this approach can facilitate an “awake-like” recording environment in an asleep patient [26].

Another novel approach is low-dose ketamine-mediated conscious sedation. A recent trial in Parkinson’s patients found that adding low-dose ketamine during MER mapping preserved intraoperative electrophysiology and lead placement accuracy while improving patient comfort compared to the standard awake technique [28]. Although studied in movement disorder DBS, this strategy could extrapolate to epilepsy cases requiring some monitoring. Overall, these innovations reflect a move toward “the best of both worlds”—i.e., maintaining critical neurophysiological signals and safety while keeping patients asleep or comfortably sedated.

Finally, DBS itself is expanding to new targets and indications in epilepsy, which influences anesthetic considerations. While the ANT remains the primary FDA-approved target for focal epilepsy, recent studies have broadened to the CMT for generalized seizures and the hippocampus for mesial temporal lobe epilepsy [3]. These targets often do not require the fine-grained MER mapping used in movement disorders, further supporting the feasibility of performing them under GA. For example, asleep, robot-assisted implantation of CMT-DBS has been reported as safe and accurate. Wang et al. (2023) described 10 drug-resistant epilepsy patients undergoing frameless robot-guided CMT lead placement entirely under GA (no MER), with all electrodes precisely placed and a mean 61% seizure reduction at 1-year follow up [29]. Likewise, initial cases of hippocampal DBS have employed asleep techniques with image guidance for electrode insertion into the mesial temporal structures.

## 6. Discussion

Deep brain stimulation is an increasingly important therapy for patients with drug-resistant epilepsy, and the choice of anesthesia during DBS implantation has significant clinical implications. Key insights from our review highlight that anesthetic management in epilepsy DBS must achieve a delicate balance between patient comfort and the preservation of neurophysiological data essential for accurate lead placement. Unlike movement disorder cases that use MERs to guide electrode location, many epilepsy DBS procedures can rely on preoperative imaging and postoperative programming to verify targets. This distinction means that general anesthesia is frequently a viable and even preferable option for epilepsy patients. The use of GA (vs. awake surgery) has clear benefits: it minimizes patient stress and movement, potentially shortens operative time, and enables the inclusion of patients who might otherwise be poor candidates for awake procedures [27]. Our review found no evidence that moving to an “asleep” technique compromises surgical accuracy or outcomes when modern guidance is used. On the contrary, centers reporting asleep DBS for epilepsy have achieved seizure reductions comparable to traditional methods, without intraoperative complications attributable to anesthesia [29]. This suggests that, in appropriately selected cases, GA can be safely embraced without sacrificing efficacy.

Anesthetic drug selection plays a pivotal role in DBS success. As detailed previously, various agents can differentially affect neuronal firing and thus the utility of intraoperative recordings. For epilepsy surgeries targeting deep nuclei, like the thalamus or hippocampus, even if the MER is not strictly required, anesthetic effects on brain activity remain relevant. For instance, excessive burst suppression from anesthetics could theoretically mask electrophysiologic signs of target engagement or precipitate hemodynamic instability. The clinical lesson is that intravenous anesthetics are generally favored over inhalational agents for maintenance due to their titratability and reduced impact on deep brain firing patterns [14,26]. 

When it comes to patient outcomes and safety, the anesthetic technique can have both direct and indirect effects. The primary direct outcomes are perioperative: hemodynamic stability, airway protection, and the avoidance of anesthetic-related complications. Patients with epilepsy may have comorbid conditions that make induction and emergence tricky—for instance, some anti-seizure medications (e.g., phenytoin, carbamazepine) are enzyme-inducing drugs that accelerate the metabolism of anesthetic agents [1], and long-term anti-seizure therapy has been associated with decreased bone density [1]. These factors necessitate careful positioning and intraoperative management. However, with careful preoperative evaluation and anesthetic planning, these risks are manageable. Importantly, no strong evidence suggests that the long-term seizure outcomes of DBS are impacted by the intraoperative anesthetic. What can influence outcomes is the accuracy of lead placement—which, as discussed, can be maintained under GA with proper technique.

However, knowledge gaps remain. To date, there have been no randomized trials in the epilepsy population comparing anesthetic techniques head to head. Future studies should include prospective trials or registries comparing anesthetic techniques in epilepsy DBS—for example, evaluating any subtle differences in seizure outcomes, cognitive recovery, or electrode placement accuracy under different anesthetic regimens. Moreover, as DBS for epilepsy is still evolving, anesthesiologists must remain vigilant and adaptable—the introduction of closed-loop DBS systems or novel targets might reintroduce the need for intraoperative testing, altering the current preference for GA. While this review synthesizes the best available evidence, it is limited by the lack of large prospective trials, reliance on small studies, heterogeneity of epilepsy syndromes, and variability in anesthetic practices across centers; therefore, some recommendations may not generalize to all settings. Still, it offers a timely summary to guide future research and clinical decision-making. Ongoing research and shared clinical experience will continue to inform best practices.

## 7. Conclusions

In the present investigation, various anesthesia techniques were examined for the implantation of deep brain stimulators in the treatment of epilepsy. Our aim was to highlight the interplay between anesthetic management and surgical success in DBS, an area of growing importance as neuromodulation becomes more widely adopted for drug-resistant epilepsy. First, the field is witnessing a clear shift toward performing DBS implantation under general anesthesia or conscious sedation (“asleep DBS”) without compromising accuracy, thanks to advances like intraoperative imaging and robotic guidance [4,29]. This evolution is making DBS more accessible and comfortable for patients, an especially pertinent development for those with epilepsy who may be poor candidates for awake surgery. Second, the choice of anesthetic agents is critical—intravenous anesthetics are generally preferable for their favorable profile on neurophysiologic monitoring, whereas inhalational agents need careful dosing if used at all [14,26].

Tailoring the anesthetic plan (including potential neuromonitoring or EEG needs) in a case-by-case manner is essential for maximizing the efficacy of DBS lead placement and minimizing perioperative complications. Third, effective interdisciplinary collaboration and planning are fundamental: the best outcomes are achieved when neurosurgeons, anesthesiologists, and neurologists work in concert to manage the unique challenges of these cases, from patient selection and preoperative medication management to intraoperative adjustments and postoperative care.

In our review, we identified that proactive strategies (such as having rescue medications ready for seizures, using BIS to guide sedation depth, and planning for postoperative analgesia and nausea control) can significantly enhance patient safety and comfort during DBS procedures [13,26].

In this narrative review, we examined various anesthesia techniques for DBS implantation in epilepsy; overall, our analysis indicates that general anesthesia can be safely used for DBS in DRE patients without compromising targeting accuracy or seizure outcomes. Intravenous anesthetic regimens (e.g., propofol-based TIVA) are preferred due to their minimal interference with neurophysiological monitoring, while careful perioperative planning by a multidisciplinary team helps mitigate risks and optimize patient comfort and safety. By building on the knowledge gaps identified, the medical community can strive to establish evidence-based guidelines for anesthetic management in DBS for epilepsy. Such efforts will undoubtedly enhance the therapeutic impact of DBS, enabling it to benefit a broader population of patients in the years to come.

## Figures and Tables

**Table 1:** Effects on MER and the benefits and drawbacks of pharmacological interventions associated with DBS.

Drug Name	Drug Class	Effect on MERs	Benefits	Drawbacks
Propofol	Direct GABA A activator [19]	Attenuation of MER signaling [16] Can mask HFOs [16]	Rapid onset, short acting, and commonly used	Dyskinetic effects and the abolition of tremors [20]
Dexmedetomidine	Selective alpha-2 adrenoreceptor agonist	Low dose; does not impact MERs Continuous infusion; lower overall neuron firing [18]	Ideal drug for DBS	Hypotension, tachycardia, and paradoxical agitation at high doses [17]
Sevoflurane and Isoflurane	Inhalational general anesthetics	Complete suppression of neuronal activity in the ANT [14], but conclusions cannot be drawn because of limited studies in epileptics	Sevoflurane and other inhalational anesthetics have not been studied enough to draw conclusions; small studies have shown alteration in MERs, and hence, they are avoided [6]	
Remifentanil and Fentanyl	Opioids	Not completely understood, but evidence of a reduction in the amplitude of action potentials and a modulation of GABA neurons [21]	Not well studied	Rigidity, bradykinesia [18], and suppression of tremors [22]
Benzodiazepines	Direct GABA agonists	Abolish MERs [18]	Amelioration of tremor [13] in PD; effects in epilepsy not well studied	Induce dyskinesias [6]. Affect intraoperative monitoring

## Data Availability

Not applicable.
